# Gastrojejunostomy without partial gastrectomy to manage duodenal stenosis in a dog

**DOI:** 10.4102/jsava.v86i1.1285

**Published:** 2015-12-09

**Authors:** Johannes J. Nel, Cornelius J. du Plessis, Gert L. Coetzee

**Affiliations:** 1 Department of Companion Animal Clinical Studies, University of Pretoria, South Africa; 2 Fourways Veterinary Hospital, Bryanston, South Africa

## Abstract

A nine-year-old female Rottweiler with a history of repeated gastrointestinal ulcerations and three previous surgical interventions related to gastrointestinal ulceration presented with symptoms of anorexia and intermittent vomiting. Benign gastric outflow obstruction was diagnosed in the proximal duodenal area. The initial surgical plan was to perform a pylorectomy with gastroduodenostomy (Billroth I procedure), but owing to substantial scar tissue and adhesions in the area a palliative gastrojejunostomy was performed. This procedure provided a bypass for the gastric contents into the proximal jejunum via the new stoma, yet still allowed bile and pancreatic secretions to flow normally via the patent duodenum. The gastrojejunostomy technique was successful in the surgical management of this case, which involved proximal duodenal stricture in the absence of neoplasia. Regular telephonic follow-up over the next 12 months confirmed that the patient was doing well.

Gastrointestinal ulceration in dogs is an adverse potential effect of long-term or incorrect use of non-steroidal anti-inflammatory drugs (NSAIDs) or corticosteroids. These ulcers can range from superficial epithelial erosions to full-thickness perforating ulcers (KuKanich, Bidgood & Knesl 2012). Smaller non-perforating gastrointestinal ulcers can often be treated with antacids, histamine H_2_-receptor agonists, proton pump inhibitors, anti-ulcer (cytoprotective) drugs and synthetic prostaglandin E_1_ (PGE_1_) analogues (Dowling 1995; Henderson & Webster 2006). However, in cases associated with perforation or when gastric outflow obstruction is caused by stenosis secondary to chronic ulceration, surgical intervention is usually indicated (Fossum & Hedlund 2003; Stanton & Bright 1989). Perforating gastrointestinal ulcers are generally treated by full-thickness excision. Pyloric stenosis and gastric outflow obstruction are reported complications in the gastrointestinal tract secondary to gastric ulceration and several surgical procedures have been described to treat this condition (Imtiaz *et al*. 2012). The choice of surgical procedure for treating pyloric outflow obstruction is determined by the cause, site and size of the obstruction and the surgeon’s preference (Papageorges, Breton & Bonneau 1987). The surgical techniques available for treatment of outflow obstruction include pyloromyotomy, pyloroplasty, Y-U pyloroplasty (Y-U antral advancement flap), Roux-en-Y gastroenterostomy, pylorectomy with gastroduodenostomy (Billroth I procedure) and, rarely, pylorectomy with gastrojejunostomy (Billroth II procedure).

In human surgery, a palliative gastrojejunostomy without partial gastrectomy was described as early as 1881, when Wolfer performed the first successful palliative gastrojejunostomy whilst operating on a case of pyloric carcinoma (Robinson 1960). In 1884, Ludwik Rydygier performed a gastrojejunostomy for an obstructing duodenal ulcer to provide a bypass for gastric contents (Weil & Buchberger 1999). Gastrojejunostomy without partial gastrectomy is used infrequently in veterinary science and mostly palliatively for inoperable or metastatic gastric lesions to improve quality of life temporarily (Papageorges *et al*. 1987; Withrow, Vail & Page 2013).

This case study describes the successful use of a palliative gastrojejunostomy in the treatment of duodenal stricture in the absence of neoplasia.

## Case history

A nine-year-old sterilised female Rottweiler was referred to a small-animal specialist referral hospital with a history of abdominal pain, tachycardia, pyrexia and melaena. Four months earlier, the dog had been treated for a perforated gastric ulcer that healed uneventfully after surgical excision. The patient had been treated with various NSAIDs and occasional corticosteroids during the previous 4 years of her life as part of medical management of elbow dysplasia and osteoarthritic pain in the stifle joint.

On presentation, the patient was weak, with pale mucous membranes, tachycardia and a normal body temperature. Blood chemistry showed elevated urea levels and decreased levels of albumin, total protein, alkaline phosphatase, alanine aminotransferase and creatinine. Electrolytes were within the normal parameters and the packed cell volume was 16%.

An ultrasonographic examination revealed regional hyperechoic mesentery and focal peritoneal effusion in the right cranial abdominal quadrant and a presumptive diagnosis of peritonitis secondary to a suspected perforated gastroduodenal ulcer was made.

The patient was stabilised and prepared for an exploratory coeliotomy. Pre- and post-operative treatment involved the use of sucralfate (Ulsanic, Aspen Pharmacare) at 1 mg/kg PO tid, amoxicillin–clavulanate (Augmentin, GlaxoSmithKline) at 12.5 mg/kg IV tid, metronidazole (Flagyl, Sanofi-Aventis) at 15 mg/kg IV bid, omeprazole (Nexium, AstraZeneca) at 1 mg/kg IV oid and buprenorphine (Temgesic, Reckitt Benckiser Healthcare) at 0.02 mg/kg SC tid.

The patient was induced with propofol (Propofol Fresenius Vail, Fresenius Kabi) at 4 mg/kg IV, an endotracheal tube was placed and anaesthesia was maintained with isoflurane (Forane, Abbott Laboratories). The patient was placed in dorsal recumbency and the entire abdomen was prepared for aseptic surgery. A ventral midline skin incision was made from the xiphoid process to the pubis. During surgery, a 1-cm full-thickness perforation of the proximal duodenum was found approximately 2 cm oral to the major duodenal papilla. There were extensive chronic adhesions present between the liver, stomach and proximal duodenum, which were broken down carefully. The scar from the previous surgery was visible approximately 4 cm oral to the area of ulceration. The duodenal ulcer was identified and a full-thickness resection was performed. The excised tissue sample was submitted for histopathological examination. The abdomen was flushed several times with large volumes of lukewarm lactated Ringers solution (Sabax Ringers lactate, Adcock Ingram Critical Care) and the abdominal cavity was closed routinely, using 0 polydiaxonone (CliniSolv, Clinisut, Port Elizabeth) to suture the linea alba, 3-0 polydiaxonone (CliniSolv, Clinisut) for the subcutaneous layer and 3-0 monofilament nylon (CliniLon, Clinisut) for the skin closure. The patient was kept in the intensive care unit for 5 days and then discharged.

Histopathology confirmed the presence of chronic ulceration with no evidence of malignancy. Ten days after the operation, the dog stopped eating and presented with intermittent vomiting. The clinical examination was unremarkable. Abdominal ultrasound examination revealed a grossly distended stomach filled with speckled hypoechoic fluid and food particles. There was no obvious transit of fluid or food passing through the duodenum, despite mild gastric contractions being present. A presumptive diagnosis of pyloric outflow obstruction secondary to stenosis at the previous surgical site in the proximal duodenum was made and an explorative coeliotomy was performed.

Enrofloxacin (5 mg/kg SC) (Baytril, Bayer) and buprenorphine (0.02 mg/kg IV) (Temgesic, Reckitt Benckiser Healthcare) were given pre-operatively. The patient was induced with propofol (Propofol Fresenius Vail, Fresenius Kabi) as described earlier, an endotracheal tube was placed and anaesthesia was maintained with isoflurane (Forane Abbott Laboratories). The patient was placed in dorsal recumbency and prepared for aseptic abdominal surgery as described earlier.

**Figure 1:**
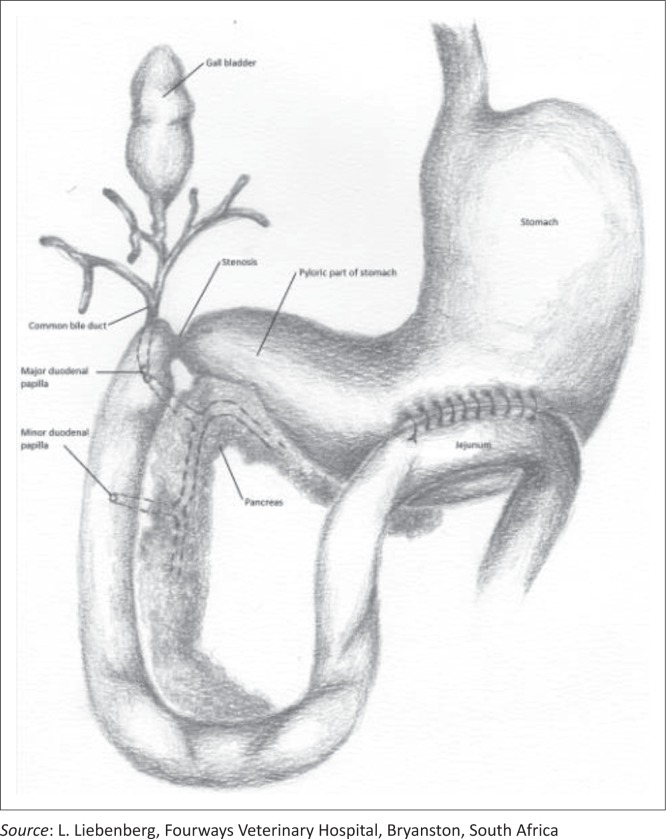
Schematic representation of the stomach, duodenum and proximal jejunum, indicating area of stenosis and area of gastrojejunostomy.

A ventral midline skin incision was made from the xiphoid process to the pubis. The stomach was distended with fluid and the presence of a stenosis at the previous surgical site in the proximal duodenum was confirmed. Macroscopically the pancreas appeared normal and the gallbladder emptied into the proximal duodenum with gentle digital pressure, confirming patency of the common bile duct. During the operation, a palliative gastrojejunostomy without partial gastrectomy was decided on. This intervention could provide a bypass for the gastric contents directly into the proximal jejunum via a new stoma yet still allow bile and pancreatic secretions to flow normally via the patent duodenum to the jejunum. A relatively avascular area of the stomach wall was identified between the lesser and greater curvature of the pyloric antrum (Figure 1). A loop of proximal jejunum, just distal to the duodenum, was attached to the visceral surface of the stomach with stay sutures. Full-thickness, 5-cm-long longitudinal incisions were made into the stomach wall and the opposing jejunal lumen. The resulting openings were sutured to create a luminal connection between the jejunum and the stomach. The mucosa and submucosa of the stomach were first sutured to the corresponding layers of the jejunum in a continuous pattern using 3-0 polydiaxonone (CliniSolv, Clinisut), followed by a continuous suture pattern (Lembert) to connect the serosa and muscularis layers of the now-opposed organs. The abdomen was flushed several times with large volumes of lukewarm lactated Ringers solution (Sabax Ringers lactate, Adcock Ingram Critical Care) and the abdomen was closed routinely as described earlier.

The patient was admitted to the intensive care unit and treatment included continued use of sucralfate (Ulsanic, Aspen Pharmacare) at 1 mg/kg PO tid, omeprazole (Nexium, AstraZeneca) at 1 mg/kg IV oid buprenorphine (Temgesic, Reckitt Benckiser Healthcare) at 0.02 mg/kg SC tid, and metoclopramide (Clopamon, Aspen Pharmacare) at 0.25 mg/kg SC tid.

On the third day post-operatively, the patient developed pyrexia that persisted for about 12 hours before the temperature returned to normal. Small amounts of water were given the day after surgery and the patient was observed for vomiting. Post-operative feeding commenced 24 hours after the surgery, initially with approximately 25% of the daily caloric requirement. The volume fed was gradually increased over the following 5 days. A commercially available canine prescription diet was liquefied to make a smooth paste and syringe fed to the patient. The frequency of feeding was gradually increased to five times a day. The dog’s appetite was initially poor, although she readily took food when syringe fed. The patient was discharged after 7 days in hospital. Enrofloxacin (5 mg/kg orally, once a day) (Baytril, Bayer) was prescribed for 7 days and omeprazole (1 mg/kg orally, once a day) (Losec MUPS, AstraZeneca) indefinitely. Her appetite slowly increased to near normal about 6 weeks after surgery.

Regular telephonic feedback over the next year confirmed that the patient was doing well, to the point where the caloric intake had to be reduced owing to excessive weight gain that exacerbated the osteoarthritic pain.

## Discussion

Gastroduodenal ulceration in dogs is a debilitating and potentially fatal condition that needs aggressive medical treatment and, occasionally, surgical intervention (Stanton & Bright 1989). The administration of NSAIDs or corticosteroids to dogs, although effective, has been associated with various adverse events, including gastrointestinal ulceration (Boston *et al*. 2003; Dowling 1995; Forsyth *et al*. 1998; KuKanich *et al*. 2012; Lascelles *et al*. 2005; Mathews 2000). Studies have demonstrated a significant increase in risk if different types of NSAID are used concurrently, if NSAIDs are administrated at higher dosages than approved and when NSAIDs are used in conjunction with corticosteroids (Case, Fick & Rooney 2010; KuKanich *et al*. 2012; Lascelles *et al*. 2005; Stanton & Bright 1989).

Gastric and duodenal ulceration can often be treated successfully with medical intervention alone; surgery is uncommonly needed and reserved predominantly for complications or refractory disease such as uncontrolled haemorrhage, acute perforation or gastric outflow obstruction (Tobias & Johnston 2012). Perforating gastrointestinal ulcers are generally treated by full-thickness excision (Tobias & Johnston 2012). Pyloric and duodenal stenoses are described complications secondary to deep ulceration that heals by scar tissue formation rather than by re-epithelialisation (Fossum 2013). Other, more common causes of pyloric stenosis are chronic antral mucosal hypertrophy and functional pyloric stenosis, mostly seen in young, male dogs of brachycephalic breeds (Peeters 1991). Polyps are adenomatous proliferations that can potentially cause outflow obstruction (Fossum 2013). Neoplastic lesions in the antrum or pyloric area are less common than in humans, but do occur in dogs and can potentially lead to gastric outflow obstruction (Ahmadu-Suka *et al*. 1988).

Several surgical procedures have been described to treat gastric outflow obstruction and depend, to some degree, on the cause and severity of the obstruction (Imtiaz *et al*. 2012). All the techniques aim to increase the diameter of the pylorus or to remove the cause of the obstruction, thereby correcting gastric outflow. These procedures include Fredet– Ramstedt pyloromyotomy, Heineke–Mikulicz pyloroplasty, Y-U pyloroplasty, Roux-en-Y gastroenterostomy, rarely pylorectomy with gastroduodenostomy (Billroth I procedure) and, very rarely, pylorectomy with gastrojejunostomy (Billroth II procedure) (Ahmadu-Suka *et al*. 1988; Fossum 2013; Hoya, Mitsumori & Yanaga 2009; Hutchinson & Kiriluk 1960; Imtiaz *et al*. 2012; Papageorges *et al*. 1987; Tobias & Johnston 2012; Walter, Matthiesen & Stone 1985).

Fredet–Ramstedt pyloromyotomy is a simple procedure during which a longitudinal incision is made through the serosa and muscularis layers, without penetrating the mucosal layer. This allows the mucosa to bulge into the incision site, thereby increasing the luminal diameter. The procedure is usually reserved for benign obstruction in the absence of neoplasia, but often provides only temporary relief, as healing of the incision can lead to scar formation, which again reduces the lumen diameter (Fossum 2013; Tobias & Johnston 2012). In Heineke–Mikulicz pyloroplasty a longitudinal incision is made in the pyloric area and closed in a transverse manner, thereby increasing the pyloric lumen diameter. This procedure is usually reserved for benign obstruction in the absence of neoplasia. It is easy to perform and allows biopsy samples to be taken at the same time (Fossum 2013).

The Y-U pyloroplasty (Y-U antral advancement flap) involves creating a Y-shaped incision in the pyloric area and advancing the pyloric antrum flap into the region of the pyloric sphincter to create a U-shaped closure (Fossum 2013). The procedure allows good exposure of the pyloric mucosa for visual inspection and biopsy sampling whilst simultaneously increasing the lumen diameter of the outflow tract. Y-U pyloroplasty is usually used for treatment of non-inflammatory, non-malignant causes of outflow obstruction that are not related to an ulcer (Imtiaz *et al*. 2012). Pylorectomy with gastroduodenostomy (Billroth I procedure) involves resection of the pylorus and an end-to-end anastomosis of the duodenum and the stomach and is usually reserved for animals with pyloric outflow obstructions secondary to neoplasia, ulceration of the outflow tract and, in some cases, pyloric hypertrophy that cannot be treated by previously mentioned pyloroplasty techniques (Bühner *et al*. 1988; Ehrlein *et al*. 1987; Fossum & Hedlund 2003; Hutchinson & Kiriluk 1960; Tobias & Johnston 2012; Walter *et al*. 1985). It is a challenging procedure and a detailed knowledge of the relative anatomy of the bile duct, pancreatic ducts and vascular supply to the area is critical (Tobias & Johnston 2012).

Pylorectomy with gastrojejunostomy (Billroth II procedure) is a surgical bypass procedure in which the distal portion of the stomach and a part of the duodenum are resected. Closure of the resulting openings are followed by a side-to-side anastomosis of a jejunum loop to the greater curvature of the stomach (Ahmadu-Suka *et al*. 1988; Tobias & Johnston 2012). The Billroth II procedure is usually reserved for neoplastic lesions that require partial or complete excision of the duodenum, precluding end-to-end anastomosis of the pylorus to the duodenum. This procedure is commonly performed in humans with gastric and duodenal ulcers and distal gastric tumours (Thompson 1977). However, in dogs the procedure is performed rarely, mainly owing to the relatively low incidence of gastric tumours compared to humans and the high post-operative morbidity reported in the human literature (Ahmadu-Suka *et al*. 1988). Although Billroth I or II procedures provide immediate relief of gastric outflow obstruction and clinical improvement in the early post-operative period, the procedures require extensive surgery and are often associated with minimal survival advantage and a poor prognosis, especially in the case of Billroth II (Fossum & Hedlund 2003).

A Roux-en-Y gastroenterostomy involves a partial gastrectomy. The cut edge of the duodenum is closed with two-layer inverting sutures. The jejunum is transected approximately 30 cm distal to the ligament of Treitz (the ligament that connects the duodenum to the diaphragm). The distal cut end of the jejunum is anastomosed end-to-end to the gastric remnant. The proximal cut end of the jejunum is anastomosed to the side of the mid jejunum, approximately 30 cm from the gastrojejunal anastomosis (Ehrlein *et al*. 1987). When damage to the common bile duct is present, a cholecystoduodenostomy is performed. Roux-en-Y gastroenterostomy is used relatively commonly in humans after a distal gastrectomy for the treatment of gastric cancer and is preferred over the Billroth II procedure owing to a lower incidence of reflux oesophagitis (Hoya *et al*. 2009). It is currently one of the procedures of choice for gastric bypass in the treatment of obesity and diabetes mellitus type 2 in humans (Hoya *et al*. 2009; Schauer *et al*. 2003). The use of Roux-en-Y gastroenterostomy in dogs has been reported previously (Ehrlein *et al*. 1987; Hocking *et al*. 1981).

In this case study, the stenotic lesion was in the duodenum and not in the pyloric area of the stomach, ruling out the use of Y-U advancement pyloroplasty. The initial surgical plan was to perform a pylorectomy with gastroduodenostomy (Billroth I procedure) to resect the stricture. In a recent case series, 88% of dogs that underwent a Billroth I procedure survived until discharge. Anorexia, sepsis, aspiration pneumonia, pancreatitis and recurrence of the neoplasia were the leading causes of death in the 12% that did not survive. Post-operative complications were mainly due to anaemia, hypoalbuminaemia, hypotension, pancreatitis, aspiration pneumonia and septic peritonitis secondary to wound dehiscence (Eisele *et al*. 2010).

Owing to substantial scar tissue and adhesions present in the area of the proximal duodenum, visualisation of the common bile duct and the two areas where the pancreatic ducts enter the duodenum was not possible. If the common bile duct or its opening into the proximal duodenum is damaged during the procedure, a cholecystoduodenostomy or cholecystojejunostomy is required. If the pancreatic duct is inadvertently damaged or ligated during surgery, lifelong supplementation with pancreatic enzymes would be necessary post-operatively (Fossum & Hedlund 2003). A Billroth II procedure was ruled out as an option owing to the considerable post-operative morbidity reported in dogs after palliative gastrojejunostomy (Beaumont 1981). A cholecystoduodenostomy or cholecystojejunostomy is often required when performing a Billroth II procedure (Fossum 2013). In dogs, as in humans, ‘dumping syndrome’ is a common complication after gastrojejunostomy, and especially after a Billroth II procedure, and is characterised by chronic vomiting, inappetence, weight loss and diarrhoea. These symptoms are the result of rapid passage of food from the stomach into the intestine (Ahmadu-Suka *et al*. 1988). In addition, a higher incidence of stomal (anastomotic) ulceration is seen in humans after a gastrojejunostomy (Scheffel, Daskalakis & Weiner 2011). These stomal ulcers are commonly reported after pylorectomy with gastrojejunostomy, especially if the gastric antrum was not resected completely during the procedure (Thompson 1977). The residual gastric antrum produces excess gastrin, leading to excessive acid secretion and subsequent stomal ulceration at the margins of the gastrojejunostomy site, generally on the jejunal side (Ahmadu-Suka *et al*. 1988). Stomal ulceration has been described in dogs after a gastrojejunostomy procedure.

Gastrojejunostomy without partial gastrectomy, as used in this patient, allows for bypass of gastric contents into the proximal jejunum yet allows bile and pancreatic secretions to still flow normally to the jejunum via the patent duodenum. The technique was used successfully in the management of this case, which involved duodenal stricture without neoplasia, and may present a viable option as a salvage procedure when the potential post-operative morbidity excludes the use of the Billroth I and Billroth II procedures.
